# Embedding research capacity strengthening in multicountry sexual health research

**DOI:** 10.1136/bmjgh-2025-020907

**Published:** 2025-11-03

**Authors:** Elizabeth Fine, Peter Muriuki, Erin C Hunter, Lianne Gonsalves, Vanessa Brizuela, CoTSIS Study Group, Erin C Hunter

**Affiliations:** 1 School of Public Health, University of Sydney, Sydney, New South Wales, Australia; 2 Maternal and Child Well-being Unit, African Population and Health Research Center, Nairobi, Kenya; 3 Department of Population, Family and Reproductive Health, University of Ghana School of Public Health, Accra, Greater Accra Region, Ghana; 4 Department of Public Health Sciences, Clemson University, Clemson, South Carolina, USA; 5 UNDP/UNFPA/UNICEF/WHO/World Bank Special Programme of Research, Development and Research Training in Human Reproduction (HRP), Department of Sexual and Reproductive Health and Research, World Health Organization, Geneva, Switzerland

**Keywords:** Health services research, Qualitative study, Global Health

## Abstract

Research capacity strengthening (RCS) is fundamental for advancing sustainable sexual and reproductive health and rights (SRHR) research. This article examines how RCS was embedded into the CoTSIS study, a multicountry cognitive testing initiative across 19 countries based on existing frameworks and experience. We offer lessons learnt for others intending to embed RCS in multicountry research implementation.

The CoTSIS study incorporated RCS at all stages of study implementation. Capacity strengthening was incorporated in study start-up through the application and selection process of study teams, protocol co-creation and adaptation of the study tool into 15 languages. RCS was also embedded through training of study teams in cognitive interviewing techniques, ethics and sensitive research methods using a flipped classroom approach, as well as by providing ongoing support during data collection and during joint analysis and results write-up. There were several lessons learnt from this experience, such as: (1) include RCS goals and budget during study planning; (2) plan for training and needs assessments; (3) benefit from the advantages of online tools; (4) budget resources for knowledge translation activities; and (5) plan for assessing sustained capacity strengthened.

By embedding specific and intentional RCS goals and activities, multicountry studies can contribute to long-lasting and sustainable RCS, encouraging power shifts and country ownership.

**Summary box**

- Practical tips for embedding research capacity strengthening activities are important for global sexual health researchers intending to strengthen research capacity.
- Previous publications have identified the need for intentional and explicit goals and activities to ensure intentionality, impact and sustainability beyond the lifespan of the study.
- While learning by doing is important and key in strengthening research capacity, it is not sufficient to ensure long-lasting change.
- We provide five lessons learnt from embedding research capacity strengthening activities: including specific goals in study planning, assessing needs for training and implementing training, benefiting from using online tools, including funds for dissemination of study results, and planning for assessing sustainability of skills built.

## Introduction

 Research capacity strengthening (RCS) aims to provide individuals and institutions with the ability to conduct independent and sustainable research.^1^,^6^ The HRP Alliance is an initiative for RCS part of the UNDP/UNFPA/UNICEF/WHO/World Bank Special Programme of Research, Development and Research Training in Human Reproduction at the WHO (HRP/WHO).^7 8^ The HRP Alliance aims to build a critical mass of sexual and reproductive health and rights (SRHR) researchers and supports multicountry research using a localised approach through seven regional RCS hubs.^7^,^9^

Between 2020 and 2023, the HRP Alliance supported an HRP/WHO-led multicountry study using cognitive testing (CoTSIS study) to refine a global questionnaire to collect data on sexual practices and experiences. The CoTSIS study protocol, which included activities aimed at strengthening research capacity and main results have been described in detail elsewhere.^10^,^12^ Building on past experiences and existing evidence, the CoTSIS study intentionally incorporated RCS activities throughout.^13^,^17^ There is ample support for embedding RCS activities into health research as an opportunity to accelerate the improvement of health outcomes globally.^514 18^,^22^ Embedded RCS has already been reported to be beneficial both to teams’ research capacity and the quality of research outputs.^5 14 23^ There are also examples reporting that research capacity is strengthened through participation in research projects, where researchers have ‘learned by doing’,^13 21 24 25^ and others where explicit activities aimed to strengthen research capacity have been included together with specified goals.^25 26^ Given the support for embedding RCS and especially as it relates to sexual health research, it has become important to document successes and lessons learnt to enhance benefits of future initiatives. In this practice paper, we describe the approach and lessons learnt in embedding RCS in a sexual health multicountry research project.

### Embedded research capacity strengthening

The CoTSIS study was implemented in 19 countries across all WHO regions through an iterative process consisting of three waves of cognitive testing.^10 11^ In-country data analyses were conducted by individual study teams, followed by joint analysis meetings for cross-country analysis to revise the tool and inform the next wave of data collection. HRP/WHO led cross-country coordination, including training study teams on protocol implementation. The HRP Alliance supported the call for proposals through their research impact grants scheme, selection of research collaborators, submission to ethics review committees and additional training needs as they arose throughout project implementation and results write-up. The entire study was coordinated remotely; trainings, all-team meetings, seminars and other online events were held at different times to accommodate time differences between countries (spanning GMT −8 to GMT +11). Meetings were held primarily in English, although live interpretation by members of the coordination was available in French, Portuguese and Spanish. On-site monitoring, site visits and in-person meetings were left to local teams and principal investigators (PIs) to conduct.

#### Research capacity strengthening approach

**Figure 1:**
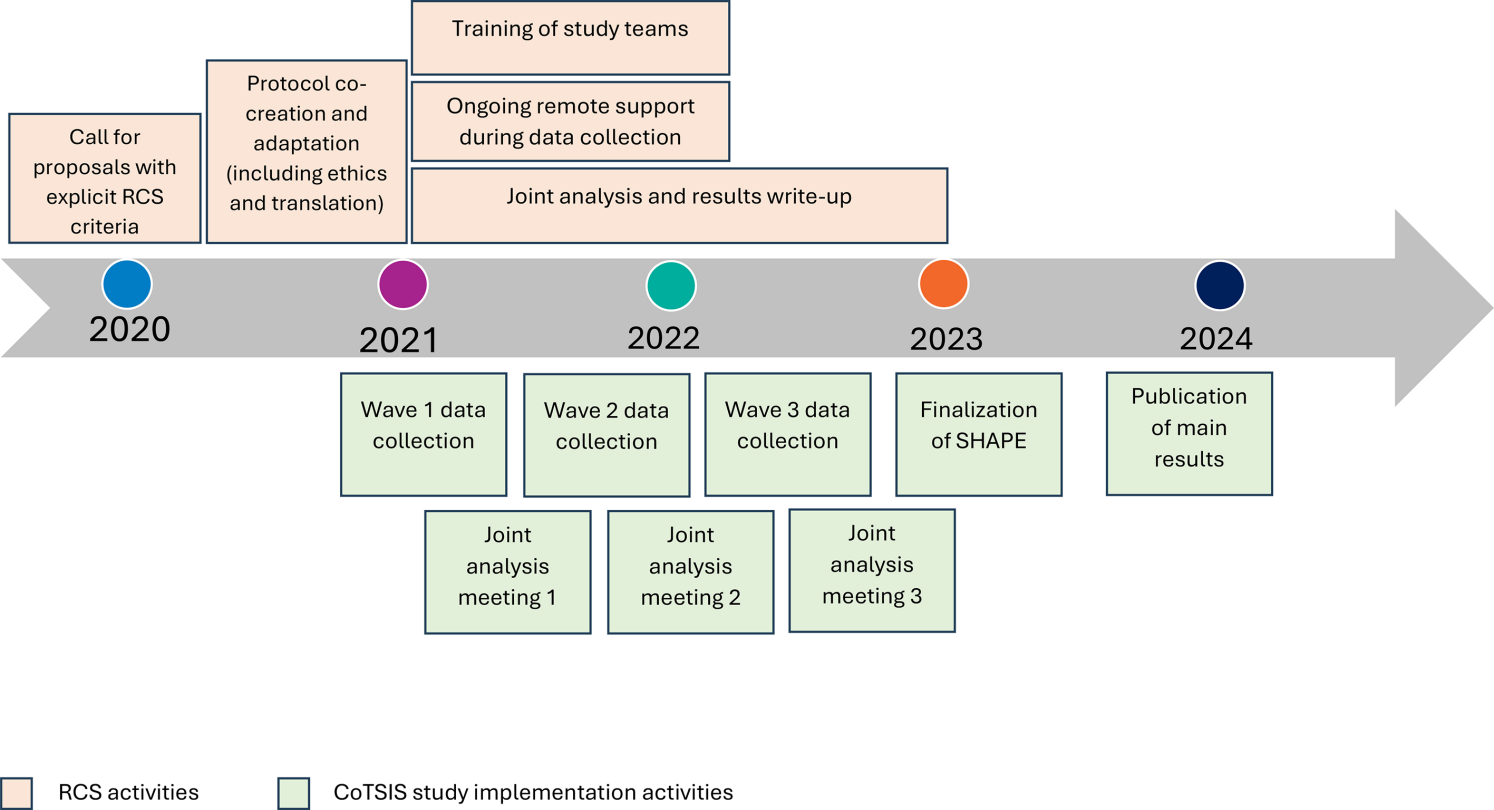
Flowchart depicting timeline of embedded RCS activities according to CoTSIS study timeline. RCS, research capacity strengthening; SHAPE, Sexual Health Assessment of Practices and Experiences.

The CoTSIS study used RCS strategies that could provide long-lasting benefits in response to calls for power shifting in global health through a series of actions and trainings.^14 27 28^ The approach used for embedded RCS drew on existing frameworks and learnings from earlier HRP multicountry research.^13^,^1729^ The specific activities implemented are detailed below. Figure 1 illustrates the timeline of RCS activities as they correlate with CoTSIS study implementation.

##### Application and selection process

In late 2020, the HRP Alliance issued a call for proposals for localising a core, master protocol for the CoTSIS study. Submissions were assessed by a panel of internal (HRP/WHO) and external experts based on scientific merit, budget, timeline, feasibility and team capacity. Selected teams needed to: (1) be embedded within academic institutions, non-governmental organisations, national health/research institutes or ministries of health; (2) be led by qualified and experienced researchers with qualitative and SRHR research experience; and (3) include women and early career researchers.^10 30^ From 33 proposals received, 20 were selected and 19 ultimately implemented the study (table 1). Four of the implementing partners were HRP Alliance regional hubs.^9^

**Table 1:** Characteristics of selected study teams and researchers (N=19 study teams; 151 researchers)

Characteristic	N (%)
Teams	**N=19**
Implementing institution	
University	8 (42)
Research institution	10 (53)
Ministry of Health	1 (5)
Average team size	8 (range: 4–15)
Gender of principal investigator*	
Woman	11 (61)
Man	7 (39)
Researchers	**N=151**
Gender of study team members*	N=136 (90)
Woman	86 (63)
Man	47 (35)
Another identity†	3 (2)
Early-career researchers*‡	80 (54)
Woman	48 (66)
Man	23 (31)
Another identity†	2 (3)

* Excludes people who did not reveal their gender identity or did not wish to be categorised.

† Includes people who identified as non-binary, non-binary/agender/trans person and other.

‡ Includes students, postdoctoral degree fellows or other types of early career/junior researchers.

##### Protocol co-creation and adaptation

Country-specific protocol adaptation was a collaborative and iterative process, which included online seminars, meetings and consultations with the coordination team to ensure alignment with local needs and context-specific issues. Adaptation also required rigorous translation of the study tool from the original English into the language most spoken in the country, for a total of 15 languages across all countries. Study teams followed a standardised translation process commonly used in survey design that included forward and back translation.^31 32^ Localised protocols incorporated detailed information regarding mandatory reporting requirements and procedures for researchers’ compliance if a participant disclosed abuse, among other situations. Local adaptation also meant ensuring appropriate consenting procedures were set in place for adolescent participants. Localisation was especially important to account for differing rules, laws and practices around sexual health (eg, abortion, LGBTIQ+ issues) across sites. Country teams were supported throughout ethics approvals responding to local norms and safeguarding participants.

##### Training of study teams

Study training was facilitated by HRP/WHO and focused on implementing the protocol, on general research skills and concepts (eg, informed consent, privacy and confidentiality) and on cognitive interviewing techniques—new to many teams—which included strategies to elicit rich responses through probing and using a structured analysis framework. Additional, needs-based trainings were offered through the HRP Alliance.

For the protocol training, we used a ‘flipped classroom’ model, where study team members engaged in independent learning through prerecorded training videos and activities, followed by virtual live sessions for reviewing key concepts, discussions and interactive activities such as role-playing. Teams were encouraged to record practice interviews for feedback from HRP/WHO. Each live training session was followed by a brief survey to assess knowledge and skills gained and level of confidence to implement the study protocol.

All training materials were stored on an internal study website, serving as a central repository for access throughout study implementation, and for teams using a train-the-trainer approach for their larger teams. Often, early career researchers and students led or supported the cognitive interviews, further providing opportunities for learning-by-doing.

To explore supplemental training needs to be offered through the HRP Alliance, at the start of the study we verbally asked all PIs to let us know if their teams required additional support. We also added questions to the surveys following each protocol training session to gauge any training needs. Through these processes, teams requested training to help navigate local mandatory reporting requirements, as well as local sensitivities and sociocultural norms surrounding topics such as abortion, sexual orientation and gender identity. Based on these requests, we added dedicated time to discuss issues relating to mandatory reporting procedures using interactive scenarios of participants disclosing distressing or concerning experiences, where we asked teams to identify how they would respond, whether reporting was required and, if so, how to proceed. Through these activities, teams discussed the differences in procedures across countries, how they had managed these situations in the past and, most importantly, where additional refinement of the procedures was required. Further, we also allotted time to discuss how to deal with participants reporting experiences of sexual violence. Based on additional needs identified early on, the HRP Alliance supported the development and delivery of a training on values clarification and attitude transformation (VCAT) by the hub at African Population and Health Research Center in Kenya.^9^ VCAT training was conducted to broaden research teams’ sensitivity and to help them examine personal attitudes that might influence data collection and interpretation, which could adversely impact the study outcomes. A total of 77 researchers from 17 country teams, including data collectors, participated in the VCAT trainings.

### Ongoing support during data collection

The HRP/WHO team led debriefing meetings with each team after the first two interviews were conducted to review the analysis framework and probing strategies, ensure sufficient details were being captured and discuss any issues during implementation. Moreover, weekly emails were sent to country teams throughout data collection to provide updates, share news or request information, which also provided opportunities for engagement and support throughout. We also coordinated regular online meetings, particularly at the start of the project, which allowed for team building among and between the different groups, especially given the fully remote nature of the project. These meetings also helped to address questions and concerns, share experiences and troubleshoot for issues arising during protocol adaptation, recruitment of participants and data collection.

### Joint analysis and results write-up

Cross-country data analysis of the 645 cognitive interviews was completed collaboratively among all research study teams through joint meetings. All study team members, including early-career researchers, were invited to attend and contribute to these meetings fostering shared learning. This mechanism also ensured that modifications to the tool following each wave of data collection were reflective of the findings emerging from the different contexts.

Teams were encouraged to plan for country-specific data analysis, manuscript writing and result dissemination activities, important markers of research capacity and excellence.^28^ Ideas for cross-country analyses were collected by the coordination team who consolidated similar ideas and encouraged teams to work together in developing and sharing a proposal. These strategies aimed to facilitate collaboration between sites and promote opportunities for leadership on data analysis. Teams were encouraged to have students and early-career researchers lead the development of manuscripts with support from the senior researchers. Mentorship throughout the scientific writing process is one way to ensure transfer of skills from senior to early-career researchers during implementation of research studies.^29 33^ An example of this is an ongoing analysis by two doctoral students from the Kenya study team who are now leading a paper with mentorship from senior researchers in their institutions and the HRP/WHO coordination team, and similar others for completing country-specific analyses.

We agreed that cross-country publications would be written on behalf of the CoTSIS Study Group to promote equitable authorship. The group is composed of 161 researchers including PIs, co-investigators, coordinators, the HRP/WHO coordination team, study steering group and other team members meeting International Committee of Medical Journal Editors (ICMJE) criteria.^34^

#### Lessons learnt

The CoTSIS study provided opportunities to more systematically and intentionally consolidate and solidify lessons learnt. For example, we initially planned to target only PIs in trainings and study meetings under the assumption that this would facilitate a more streamlined approach; however, we quickly realised that expanding access to all research team members would be more beneficial. Participation also encouraged engagement and ownership and exposed all researchers to integral components of conducting multicountry research, including study planning and management.^13 14 35^ This also offered team members who were closely engaged in the data collection with opportunities to learn about cognitive interviewing, including analysis and interpretation of data, and to take on leadership roles in study coordination and results write-up within their sites and across the study group. These joint sessions offered a space for sharing challenges and gave rise to the development of VCAT. Our successes, as well as missed opportunities and shortcomings in integrating RCS in the CoTSIS study, inform several lessons learnt.

##### Include research capacity strengthening goals during study planning

Research teams were selected based on several criteria, which were clearly indicated in the application form. This strategy helped ensure that applicants were responsive to the RCS goals of the study and to select teams that were most likely to succeed, and benefit, from participating. While we were successful in ensuring all study teams included women and early-career researchers, evaluation of RCS was not included as a study objective, meaning that it was not budgeted for or planned. Therefore, it was not measured and we were unable to assess whether team capacity was, in fact, strengthened and that any capacity strengthened was sustained over time. Furthermore, given no RCS objectives were included, it was up to country teams to decide on how and what RCS activities to do which also had implications for budgeting and resource allocation. Future multicountry studies should consider including RCS as a study objective and to support teams in developing them. Teams should also be supported in monitoring and evaluating the activities to ensure that efforts at strengthening individual and institutional capacities are captured.^14 15 17^ Relatedly, study teams would benefit from pre-protocol and budget development discussions that would allow for planning realistic resources to complete all RCS-related activities. We acknowledge that this may not always be possible or feasible, but it could be instrumental to the success of the study implementation.

##### Plan for training and needs assessments

The CoTSIS study succeeded in contributing to RCS by training study teams in cognitive interviewing, which was new for many. Trainings received positive feedback and we had satisfactory engagement from all teams. Making all study materials available through the internal study website allowed for re-training; however, the usage of these materials and resources was not assessed.

Our informal approach to needs assessment was successful in identifying some training needs and allowed us to respond accordingly, with VCAT training being a key example. However, this approach left other training needs unaddressed, as we found during implementation that some teams would have benefitted from additional training on cognitive interviewing methods and data analysis. A systematic needs assessment, which has been suggested by others, may have identified these additional needs and allowed us to address them accordingly.^14^ We encourage coordinators of multicountry studies to include an individual formal needs assessment, to all study team members rather than solely asking the PI to report on behalf of the team.^13 36^ While post-training surveys allowed researchers to request additional training and teams were regularly reminded to reach out about available opportunities, few teams submitted such requests. To ensure that additional trainings are accessible, we highly recommend that future multicountry studies plan for and proactively offer re-training or specific trainings on general research skills. Unfortunately, we did not systematically explore why teams did not respond to the offerings. We suspect that the requests were too broad, creating challenges for teams to identify what training was needed or what was available to them, or team members may not have had the time or resources to attend additional trainings outside of the study-specific trainings. It is also possible that researchers did not have questions immediately following the sessions to share through surveys or that they felt uncomfortable asking for additional support.

##### Benefit from the advantages of online tools

Our experience implementing the CoTSIS study has shown the feasibility of coordinating multicountry observational studies entirely online. For this study, it was necessary to do all activities remotely due to restrictions during the COVID-19 pandemic and there were several advantages to this approach. Trainings and analysis meetings were open to all study team members, as remote convenings do not have the budgetary constraints that in-person meetings do. This offered data collectors and junior researchers the same opportunities to interact with other teams as PIs did. Additionally, remote coordination is both environmentally conscious by removing air travel and empowering by shifting much of the coordination and monitoring to local teams during implementation. Further, evidence has shown that remote training can be effective and satisfactory, especially while accompanied by mentorship.^37^ However, we were never able to have all study teams meet at the same time due to the span of time zones across research teams. There were some challenges with online training due to limited network connectivity for some team members, and active participation may have been restricted because it was online. Finally, the lack of in-person interaction hindered network building and collaboration among researchers, which may be a reason for limited engagement in the data analysis and manuscript writing phase. While there are many benefits to entirely remote coordination of multicountry studies, a model including some in-person aspects, particularly when it comes to data analysis and writing, might be more beneficial.

##### Budget resources for knowledge translation activities

Multicountry studies produce a wealth of data providing opportunities for involved researchers to strengthen their data analysis and scientific writing skills, as well as publishing resulting manuscripts and other knowledge translation materials.^28 35 38^ Despite early efforts to plan for secondary analyses when teams were still engaged, including guidance to set aside funds and time for this, most proposed analyses have either not yet progressed or are still unpublished. Advance planning was not sufficient to overcome challenges faced by researchers attempting to write up results after formal study engagement ended. This was complicated by lack of funds to cover the researchers’ time for these activities. Researchers, including students, began other projects and took on new responsibilities, leaving limited time for further data analysis and manuscript writing, a commonly reported reason for not publishing research.^39^ This has resulted in large swathes of data remaining unused and missed capacity building opportunities, particularly for students. For early-career researchers whose academic careers hinge on peer-reviewed publications, these missed opportunities can have long-lasting impacts.^39 40^

Future multicountry studies should consider incorporating funding and strategies to facilitate analysis and writing, including less resource-intense write-ups that could be used for disseminating findings at a local level and for different audiences. Examples include hosting manuscript writing workshops, retreats or writing accountability groups which have been shown to be effective methods for promoting completion and submission of scholarly publications in the health sector.^41 42^ These efforts facilitate engagement in the writing process and provide opportunities for collaboration and strengthening of writing skills, as well as for policy change. Similarly, resources should be assigned for dissemination meetings with local policymakers for results uptake. Additionally, identifying early-career researchers at the start of the project to take on data analysis and writing tasks, and support them through scholarships or small grants, would increase the research outputs produced. If appropriate for the study design, others may also consider beginning writing during the study implementation period, when study teams are actively engaged and have resources to allocate towards writing. Finally, offering use of the dataset to other students, depending on data sharing agreements made, could provide additional opportunities.

##### Plan for assessing sustained capacity strengthened

Evidence from RCS initiatives speaks to the benefit of including activities around training, explicit inclusion of early career researchers in research leadership and co-creation of research, among others.^19 42^ Further to that, learning-by-doing through participation in research studies where skills learnt are put into practice are also broadly mentioned as effective RCS mechanisms.^13 25^ While we included short assessments following each of the training sessions and provided mentorship and feedback immediately following early data collection, we did not include longer-term follow-up assessments to determine whether the benefits were sustained. Anecdotally, we know that some country teams are using cognitive interviewing techniques in upcoming studies, but we have not collected this information systematically from all study teams. Planning for continuous monitoring and sustained evaluation, which would include setting funds aside, is necessary to understand the impacts of RCS efforts and provide robust insights for others to learn from.^14 18^

## Conclusion

Our experience highlights the importance of explicit inclusion of RCS throughout the entire study process, including incorporation of RCS objectives in the study, planning for training and needs assessments, using online tools, budgeting for knowledge translation activities and planning for assessing sustainability of efforts. By embedding specific and intentional opportunities, such as those recommended above, multicountry studies can contribute to long-lasting and sustainable RCS, encouraging power shifts and country ownership.

## Data availability statement

Data sharing not applicable as no datasets generated and/or analysed for this study.
